# Physiologic left ventricular ejection efficiency assessed at the level of the aorta

**DOI:** 10.1186/1532-429X-18-S1-P138

**Published:** 2016-01-27

**Authors:** Mark Doyle, Geetha Rayarao, Victor Farah, Diane V Thompson, Ronald Williams, June A Yamrozik, Moneal Shah, Robert W Biederman

**Affiliations:** Cardiac MRI, Allegheny General Hospital, Pittsburgh, PA USA

## Background

Cardiovascular magnetic resonance (CMR) imaging has long been used to calculate the ejection fraction (EF) of the left ventricle (LV) and more recently to calculate the ventricular-vascular-coupling (VVC) ratio, both entirely measured within the LV. Here we seek to demonstrate the role of blood transmission efficiency measure within the aorta.

### Objective

To establish the nature of the linkage between aortic vascular ejection efficiency (VEE) and LV EF and VVC which may prove useful for predicting future LVEF decline.

## Methods

Vascular ejection efficiency (VEE) is proportional to the dimensionless ratio of aortic diameter to wavelength of the transmitted blood pulse. The wavelength of ejected blood is proportional to blood wave velocity divided by blood wave frequency measured using phase velocity mapping (PVM) applied to the ascending aorta. Left ventricular function and aortic PVM data were retrospectively obtained in 118 patients who underwent CMR scanning as a clinical routine. The LV end-diastolic and end-systolic volumes were measured using manually drawn contours for a contiguous stack of LV slices. The aortic area was measured from the PVM images along with the average velocity, taken as a measure of blood wave velocity (not to be confused with the more common pulse wave velocity), the end-systolic time was taken as the pulse length and aortic area instead of diameter to yield the VEE index:

VEE = Aortic Area × End systolic time / Average blood velocity

The VEE was calculated and plotted against the ventricular measures of EF and VVC.

## Results

**Figure 1:**
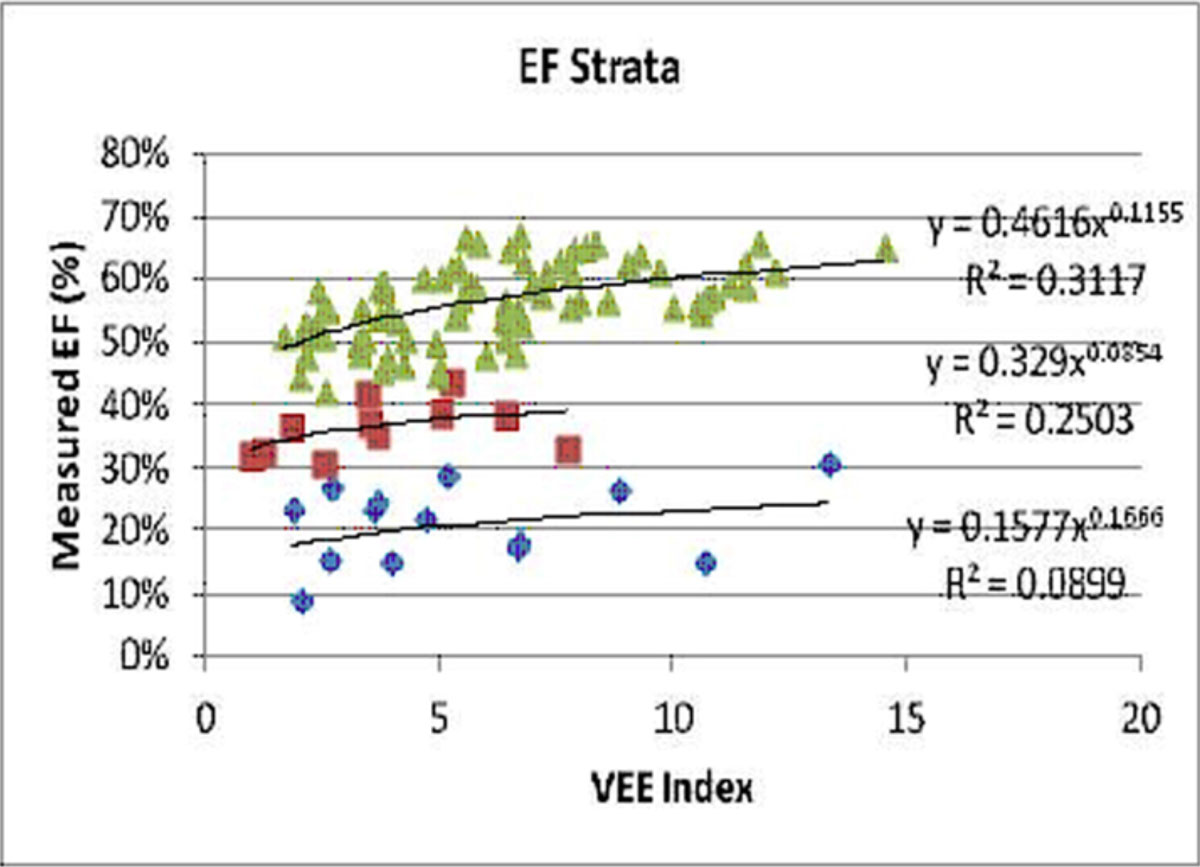
**Plot of EF vs. Aortic Efficiency Index**.

**Figure 2:**
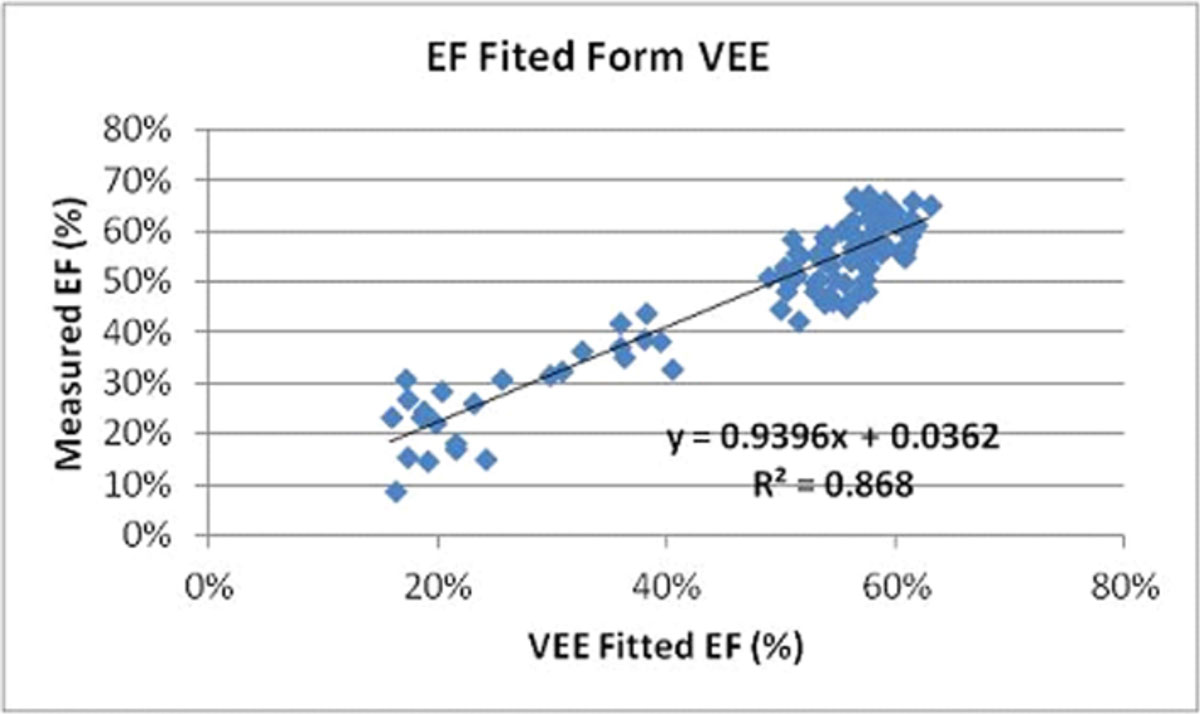
**Plot of measured vs. fitted EF**.

In the plots of EF vs. VEE patients stratified into three regions, Figure 1: upper) EF >40, middle) EF in the 30 s, and lower) EF <30). Each strata was characterized by a power relationship relating EF to VEE, Figure 1. The upper strata corresponds to patients with a VVC in the optimal working range of 0.5 to 1.2, the middle strata has VVC in the transition range 1.3 to 2.1 and the lower strata has a VVC >2.2 which is associated with heart failure. The calculated EF from the aortic transmission index measured entirely within the aorta strongly correlated with the volumetric LV measure EF (r =0.94) with a linear relationship (fitted EF = .94 VVE + .04), Figure 2.

## Conclusions

We show that vascular ejection efficiency (VEE) measured in the aorta relates to LV EF measured in the ventricle via a series of power relationships, which accommodates the precipitous decline in EF at the lower levels of VEE. VEE may permit more effective risk-stratification and early detection for patients who are near an abrupt decline in LV systolic function. To date, predictive modeling for predicting precipitous declines in cardiac function is not in existence.

